# Population dynamics of two antilisterial cheese surface consortia revealed by temporal temperature gradient gel electrophoresis

**DOI:** 10.1186/1471-2180-10-74

**Published:** 2010-03-11

**Authors:** Emmanuelle Roth, Susanne Miescher Schwenninger, Madlen Hasler, Elisabeth Eugster-Meier, Christophe Lacroix

**Affiliations:** 1 Laboratory of Food Biotechnology, Institute of Food Science and Nutrition, ETH-Zurich, 8092 Zurich, Switzerland; 2 Research Station Agroscope Liebefeld-Posieux ALP, 3003 Bern, Switzerland

## Abstract

**Background:**

Surface contamination of smear cheese by *Listeria *spp. is of major concern for the industry. Complex smear ecosystems have been shown to harbor antilisterial potential but the microorganisms and mechanisms involved in the inhibition mostly remain unclear, and are likely related to complex interactions than to production of single antimicrobial compounds. Bacterial biodiversity and population dynamics of complex smear ecosystems exhibiting antilisterial properties *in situ *were investigated by Temporal temperature gradient gel electrophoresis (TTGE), a culture independent technique, for two microbial consortia isolated from commercial Raclette type cheeses inoculated with defined commercial ripening cultures (F) or produced with an old-young smearing process (M).

**Results:**

TTGE revealed nine bacterial species common to both F and M consortia, but consortium F exhibited a higher diversity than consortium M, with thirteen and ten species, respectively. Population dynamics were studied after application of the consortia on fresh-produced Raclette cheeses. TTGE analyses revealed a similar sequential development of the nine species common to both consortia. Beside common cheese surface bacteria (*Staphylococcus equorum, Corynebacterium *spp., *Brevibacterium linens, Microbacterium gubbeenense*, *Agrococcus casei*), the two consortia contained marine lactic acid bacteria (*Alkalibacterium kapii*, *Marinilactibacillus psychrotolerans*) that developed early in ripening (day 14 to 20), shortly after the growth of staphylococci (day 7). A decrease of *Listeria *counts was observed on cheese surface inoculated at day 7 with 0.1-1 × 10^2 ^CFU cm^-2^, when cheeses were smeared with consortium F or M. *Listeria *counts went below the detection limit of the method between day 14 and 28 and no subsequent regrowth was detected over 60 to 80 ripening days. In contrast, *Listeria *grew to high counts (10^5 ^CFU cm^-2^) on cheeses smeared with a defined surface culture.

**Conclusions:**

This work reports the first population dynamics study of complex smear ecosystems exhibiting *in situ *antilisterial activity. TTGE revealed the presence of marine lactic acid bacteria that are likely related to the strong *Listeria *inhibition, as their early development in the smear occurred simultaneously with a decrease in *Listeria *cell count.

## Background

The surface of traditional smear-ripened cheeses is colonized by a complex microbial ecosystem. Its biodiversity has been investigated by identification of cultivable isolates with molecular techniques, such as Pulsed-field gel electrophoresis (PFGE), Repetitive sequence-based PCR (rep-PCR) and 16S rDNA sequencing, or with Fourier-transform infrared spectroscopy (FTIR) [1-3]. Biodiversity studies using culture independent fingerprinting techniques, such as Temporal temperature gradient gel electrophoresis (TTGE), Denaturing gradient gel electrophoresis (DGGE), Single strand conformation polymorphism (SSCP) and Terminal restriction fragment length polymorphism (T-RFLP), have revealed the presence of additional uncultivable species [4-6]. The development of the smear is a dynamic process driven by metabiosis leading to the successive growth of several microbial communities. The first microorganisms to colonize the surface are yeasts. Yeasts' deacidification properties create a favorable environment for the next populations, mainly staphylococci followed by coryneforms. These two shifts in the microbial community structure of the smear have been observed in multiple studies [6-8]. Various marine bacteria have also been detected recently on cheese surface [5,9,10]. Population dynamics of complex cheese surface ecosystems at species level have been studied by cultivation methods, but these approaches are necessarily limited by the selectivity of the cultivation media chosen. Alternatively, fingerprinting techniques can be used to generate data on main populations of such ecosystems. These methods are fast and can give a more exhaustive view of the biodiversity in cheese but they are greatly influenced by the quality of DNA extraction protocols and bias may be introduced by the PCR amplification step [11]. The main advantage of TTGE/DGGE over other available fingerprinting techniques is the possibility to identify detected species by sequencing of DNA fragments after excision from the TTGE/DGGE gel [11]. TTGE/DGGE has been applied to study dominant bacteria of dairy products, enabling detection of species accounting for at least 1 to 10% of the total flora, depending on the amplification efficiency of the PCR step for a given species [4,12].

Surface contamination of smear cheese by *Listeria monocytogenes *is of concern for the industry since listeriosis breakouts have been associated with consumption of cheese [13]. Improvements in hygienic conditions and application of safety guidelines failed to reduce the contamination frequency to an acceptable level [14]. Growth of *Listeria *on cheese surface is closely linked to the development of the surface ecosystems and is primarily supported by yeast growth, which leads to deacidification and provides nutrients for bacterial growth. *Listeria *sp. has been shown to grow easily on smear cheeses when defined ripening cultures containing *Debaryomyces hansenii*, *Geotrichum candidum *and *Brevibacterium linens *were used [15,16]. Certain complex consortia naturally developing on smear cheese surface have been shown to inhibit *Listeria *sp. *in situ *[9,15,17]. *In vitro *studies of these anti-listerial activities led to the isolation of bacteriocin-producing strains among ripening microorganisms in certain cases [18,19]. Application of the bacteriocin producing strain on artificially contaminated cheeses failed however to fully restore the inhibition [15] or disturbed the development of the smear [20]. A better knowledge of microbial biodiversity and *in situ *population dynamics is crucial to identifying species that may be involved in the inhibition. Saubusse *et al. *[21] successfully used this approach for detecting antilisterial flora naturally developing in the core of Saint-Nectaire type cheese.

The objective of the present study was therefore to investigate population dynamics of complex cheese surface consortia with respect to their *in situ *inhibition properties. Two surface consortia were isolated from commercial Raclette type cheeses. TTGE was used for assessing biodiversity of both consortia at species level. An in-house database for species-level identification of the bands appearing in the TTGE fingerprints was developed with cultivable isolates. The two complex consortia or a control flora (defined commercial culture) were then applied on freshly-produced Raclette cheeses that were artificially contaminated with *Listeria innocua*. Population dynamics and *Listeria *growth were monitored over 60 to 80 ripening days.

## Results

### Bacterial biodiversity of cheese surface consortia by cultivation - Development of a TTGE profiles database

**Figure 1:**
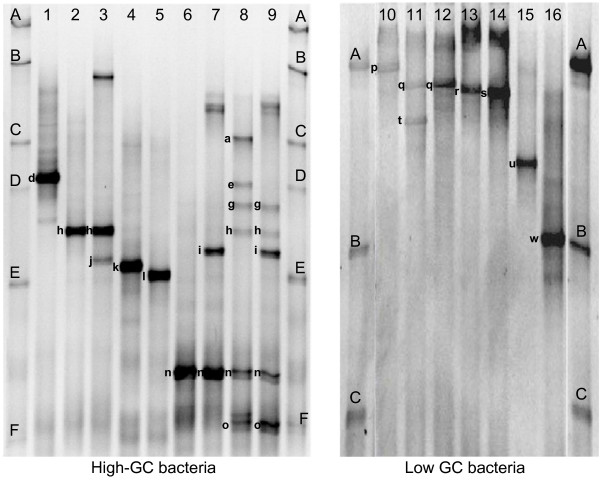
**Database for species-level identification of bands in TTGE fingerprints of complex cheese surface ecosystems**. 128 isolates from consortium F were grouped into 16 TTGE profiles corresponding to 15 species. TTGE profiles 1-9 and 10-16 were analyzed on gels optimized for the separation of high-GC bacteria and low-GC bacteria, respectively. 1, *Microbacterium gubbeenense *(band d); 2, 3, *Corynebacterium casei *(bands h, j); 4, *Brachybacterium tyrofermentans *(band k); 5, *Brachybacterium *sp. or *Arthrobacter arilaitensis *from the ladder (band l); 6, 7, 8, 9, *Brevibacterium linens *(bands a, e, g, h, i, n, o); 10, *Staphylococcus vitulinus *(band p); 11, *Staphylococcus equorum *(bands q, t); 12, *Staphylococcus equorum, Staphylococcus epidermidis *or *Facklamia tabacinasalis *(band q); 13, *Enterococcus malodoratus *(band r); 14, *Enterococcus faecium *or *Enterococcus devriesei *(band s); 15, *Enterococcus faecalis *(band u); 16, *Lactococcus lactis *or *Marinilactibacillus psychrotolerans *(band w). Ladder: A, *Lactobacillus plantarum *SM71; B, *Lactococcus lactis diacetylactis *UL719; C, *Corynebacterium variabile *FAM17291; E, *Arthrobacter arilaitensis *FAM17250; D, F, *Brevibacterium linens *FAM17309.

**Figure 2:**
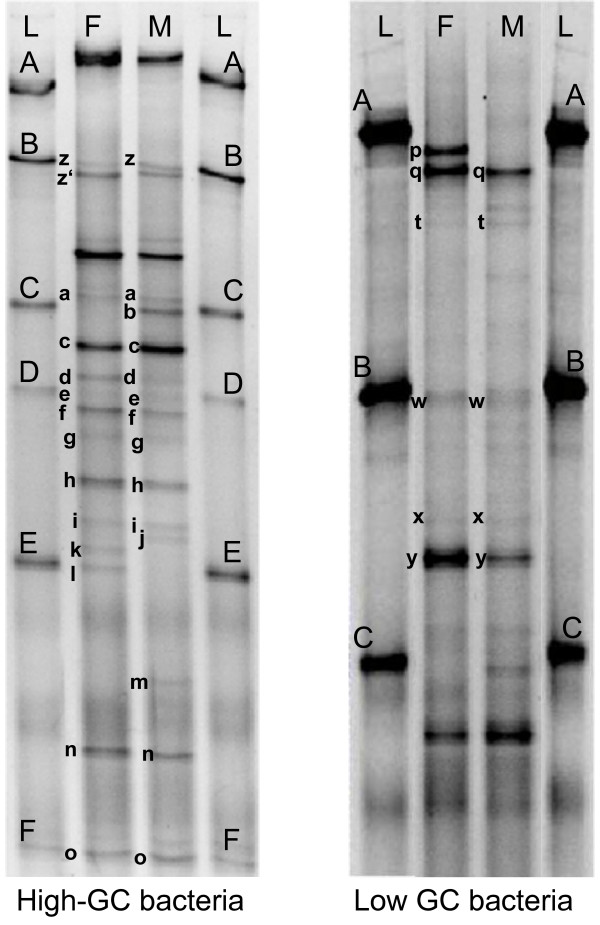
**Biodiversity of cheese surface consortia F and M by a culture independent method**. TTGE fingerprints were analyzed on two different gels (high and low GC) after total DNA extraction of cheese surface consortia. Single bands were assigned to species using the species database or by excision, cloning and sequencing (*). b, c*, *C. variabile*; d, *Mc. gubbeenense*; f*, uncultured bacterium from marine sediment; h, j, *C. casei*; k, *Br. tyrofermentans*; l, *Brachybacterium *sp.; m*, *Br. paraconglomeratum*; a, e, g, h, i, n, o, *B. linens*; p, *St. vitulinus*; q, *St. equorum*, *St. epidermidis *or *F. tabacinasalis*; q, t, *St. equorum*; w, *Lc. lactis *or *M. psychrotolerans*; x*, *Ag. casei*; y*, *Al. kapii*; z, *Lc. lactis*; z', *M. psychrotolerans*. **L**, Ladder: A, *Lb. plantarum *SM71; B, *Lc. lactis diacetylactis *UL719; C, *C. variabile *FAM17291; E, *A. arilaitensis *FAM17250; D, F, *B. linens *FAM17309.

Consortium F was serial plated on five selective and non-selective media. A total of 128 cultivable isolates were subjected to TTGE fingerprinting analysis and grouped into 16 TTGE profiles. One representative isolate of each profile was randomly selected and subjected to 16S rDNA sequencing. Isolates displaying identical TTGE profiles but isolated from different cultivation media or exhibiting different macroscopic or microscopic morphologies were also subjected to 16S rDNA sequencing analyses. The presence of 15 species was detected by cultivation, with 7 dominant species enumerated on TGYA, the medium used for the determination of total cell count (Table 1). The number of bands and the corresponding migration lengths were recorded in a database (Figure 1). A majority of species displayed TTGE profiles with a single band for all isolates. Three species showed strain variations in TTGE profiles, with some strains harboring 1 to 5 supplementary bands (Figure 1). In addition, several species had indistinguishable TTGE profiles. Profile 5 corresponded to both *Brachybacterium *sp. and *Arthrobacter arilaitensis*, profile 12 to *Staphylococcus equorum*, *Staphylococcus epidermidis *and *Facklamia tabacinasalis*, and profile 16 to both *Lactococcus lactis *and *Marinilactibacillus psychrotolerans *(Figure 1). Low-GC bacteria *Lc. lactis *and *M. psychrotolerans *could not be distinguished on low-GC gel whereas high-GC gel revealed specific bands for the two species (bands z and z', respectively, in Figure 2). The database (Figure 1) contained a total of 16 TTGE profiles corresponding to 15 species. It was used as reference for species-level identification in TTGE fingerprints obtained by the culture independent approach.

**Table 1:** Bacterial composition of cheese surface consortium F by a culture dependent method^1^

Bacterial species						Accession number^2^	Similarity (%)	Isolation media^3^	Viable count [CFU cm^-2^]	Percentage on TGYA
*Brevibacterium linens * (or *Brevibacterium aurantiacum*^4^)						GenBank:AJ315491 (GenBank:X76566^4^)	95.5-98.0 (97.8)	TGYA	7.5.10^8^	32.5%
*Staphylococcus vitulinus*						GenBank:NR_024670	99.6	TGYA	6.0.10^8^	26.0%
*Brachybacterium tyrofermentans*						GenBank:X91657	97.9	TGYA	4.5.10^8^	19.5%
*Corynebacterium casei*						GenBank:DQ361013	100.0	TGYA	1.5.10^8^	6.5%
*Microbacterium gubbeenense*						GenBank:AF263564	97.9	TGYA	1.5.10^8^	6.5%
*Marinilactibacillus psychrotolerans*						GenBank:AB083413	99.8	TGYA	1.5.10^8^	6.5%
*Brachybacterium *sp.						GenBank:AF513397	99.9	TGYA	0.7.10^8^	3.0%

*Staphylococcus equorum*						GenBank:NR_027520	98.8-99.1	MSA	3.0.10^8^	-
*Staphylococcus epidermidis*						GenBank:NC_004461	98.5	MSA	8.10^7^	-

*Facklamia tabacinasalis*						GenBank:Y17820	99.1	BP	6.10^5^	-

*Lactococcus lactis*						GenBank:NC_002662	99.5	MRS	4.10^4^	-
*Enterococcus devriesei*						GenBank:AJ891167	98.2	MRS	1.10^4^	-
*Enterococcus malodoratus*						GenBank:Y18339	99.8	MRS	2.10^3^	-

*Enterococcus faecalis*						GenBank:AJ420803	99.3	KFS	2.10^2^	-
*Enterococcus faecium*						GenBank:EU547780	100.0	KFS	6.10^1^	-

^1 ^128 isolates, i.e. ca. 25 isolates per media, were analyzed by TTGE and grouped into identical TTGE profiles. A representative isolate of each profile was identified by 16S rDNA sequencing. After the assignment of all isolates to a species, the percentage of each species on each of the five media was assessed. The cell count of a species was calculated by multiplying percentage and cell count determined for the corresponding media.

^2 ^Closest 16S rDNA sequence in the GenBank public database http://www.ncbi.nlm.nih.gov.

^3 ^Total cell count was determined on TGYA. In addition, staphylococci were enumerated on BP agar and MSA, lactic acid bacteria on MRS agar and enterococci on KFS agar.

^4 ^Given the polymorphy in the intraspecies diversity of *B. linens *(Oberreuter *et al. *[52]), strain assignation to *B. linens *or the related species *B. aurantiacum *based on 16S rDNA analysis only was considered not reliable.

### Bacterial biodiversity of cheese surface consortia by TTGE fingerprinting

Bacterial biodiversity of consortium F and M was assessed by TTGE fingerprinting of total DNA extracts, a culture independent method (Figure 2). Both consortia were analyzed on two gels, targeting the bacterial species with high-GC and low-GC content in separate runs. TTGE fingerprints of consortia F and M were composed of 18 bands each, corresponding to 13 and 10 species, respectively. Five bands could not be assigned to a known species of the database and were therefore submitted to cloning and sequencing after excision (Table 2). High similarity was found between consortium F and M with 9 common species, i.e. *Corynebacterium variabile, Microbacterium gubbeenense*, an uncultured bacterium from marine sediment (Table 2), *Corynebacterium casei*, *Brevibacterium linens*, *Staphylococcus equorum, Lactococcus lactis, Agrococcus casei *and *Alkalibacterium kapii*. Consortium F showed a higher diversity than consortium M with four additional species, *Brachybacterium tyrofermentans*, *Brachybacterium *sp., *Marinilactibacillus psychrotolerans *and *Staphylococcus vitulinus*. The species *Brachybacterium paraconglomeratum *was specific to consortium M.

**Table 2:** Identification of non-assigned TTGE bands by excision, cloning and sequencing

Band Designation^1^	Bacterial species	Accession number^2^	Similarity (%)
c	*Corynebacterium variabile*	GenBank:AJ783438	98.3
f ^3^	uncultured bacterium from marine sediment	GenBank:FJ717185	97.2
m	*Brachybacterium paraconglomeratum*	GenBank:AJ415377	96.8
x	*Agrococcus casei*	GenBank:DQ168427	100
y	*Alkalibacterium kapii*	GenBank:AB294171	97.5

^1 ^These designations are used to annotate bands from TTGE gels in figures 2 and 3.

^2 ^Closest 16S rDNA sequence in the GenBank public database http://www.ncbi.nlm.nih.gov.

^3^The 16S rDNA sequence of band f exhibited highest similarity of 94% with *Clostridiisalibacter paucivorans *(GenBank: EF026082), a bacterium that belong to cluster XII of the *Clostridium *subphylum [53].

### Population dynamics of cheese surface consortia by cultivation methods

Total cell counts and yeast counts were similar for all cheeses, independent of the surface flora applied to cheeses, i.e. consortium F, M or control flora OMK 704. Total cell counts increased from 1.2 ± 0.4 × 10^7 ^CFU cm^-2 ^to 1.2 ± 0.7 × 10^9 ^CFU cm^-2 ^within 14 days and remained stable afterwards (1.7 ± 1.0 × 10^9 ^CFU cm^-2^). Yeast counts increased from day 4 to reach 6.5 ± 0.2 × 10^6 ^CFU cm^-2 ^at day 7 and decreased afterwards by 2 to 3 log until the end of ripening. Mould counts of ca. 10^2 ^CFU cm^-2 ^were measured after 3 weeks ripening on cheeses treated with consortium F, while no moulds were detected on the cheese treated with consortium M or on control cheese. At the end of ripening, similar mould counts of ca. 10^4 ^CFU cm^-2 ^were measured on all cheeses. The pH of cheese surface increased from 5.5 ± 0.1 at day 4 to 6.8 ± 0.4 at day 7 to 10, depending on the cheese, and was constant afterwards, with mean pH of 7.2 ± 0.4.

### Population dynamics of complex cheese surface consortia by TTGE fingerprinting

**Figure 3:**
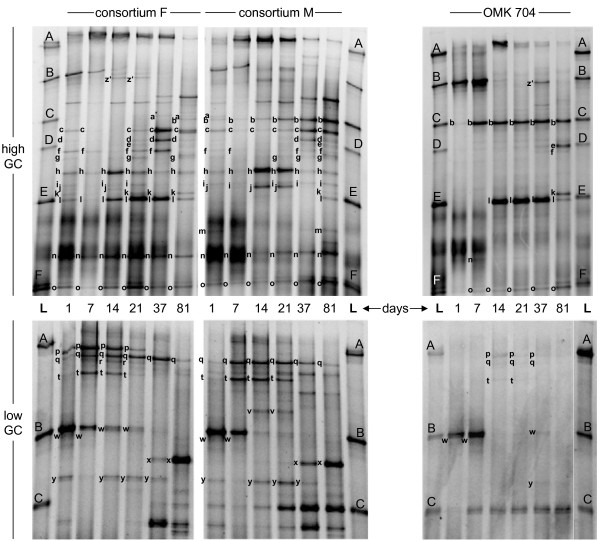
**Population dynamics of cheese surface consortia by TTGE**. TTGE analysis was carried out after total DNA extraction of cheese surfaces treated with complex surface consortium F, complex surface consortium M, or defined commercial culture OMK 704 (control cheese). Cheeses were sampled after 1, 7, 14, 21, 37 and 81 days. Each sample was analyzed on two different gels (high and low GC). Single bands were assigned to species using the database of 15 cultivable species completed by the database of 5 species identified by excision, cloning and sequencing. b, c, *C. variabile*; d, *Mc. gubbeenense*; f, uncultured bacterium from marine sediment; h, j, v, *C. casei*; k, *Br. tyrofermentans*; l, *Brachybacterium *sp. or *Arthrobacter arilaitensis*; m, *Br. paraconglomeratum*; a, e, g, h, i, n, o, *B. linens*; p, *St. vitulinus*; q, *St. equorum*, *St. epidermidis *or *F. tabacinasalis*; q, t, *St. equorum*; r, *E. malodoratus*; w, *M. psychrotolerans *or *Lc. lactis*; x, *Ag. casei*; y, *Al. kapii*; z', *M. psychrotolerans*. **L**, Ladder: A, *Lb. plantarum *SM71; B, *Lc. lactis diacetylactis *UL719; C, *C. variabile *FAM17291; E, *A. arilaitensis *FAM17250; D, F, *B. linens *FAM17309.

Population dynamics of consortium F or M were assessed at species level by TTGE fingerprinting of total DNA extracts (Figure 3, Table 3). TTGE fingerprints of day 1 cheese depict the starter culture (*Lc. lactis*) as well as the composition of the smear brines. Multiple shifts in the microbial community structure of cheeses treated with complex surface consortia F or M were observed throughout ripening. The nine species common to both consortia had similar sequential development on cheese surface. *Lc. lactis*, used as starter culture for cheese manufacture, was part of the dominant flora until day 7 and disappeared thereafter. *St. equorum *was the first species to colonize the surface within 7 days. *Al. kapii *grew on day 14 concomitant with *C. casei *and *B. linens*, followed by *C. variabile*, an uncultured bacterium from marine sediment and *Mc. gubbeenense *between day 14 and 37. *Agrococcus casei *was first detected on day 37. Other species specific to consortium F (*St. vitulinus, Enterococcus *sp., *M. psychrotolerans*, *Brachybacterium *sp. and *Br. tyrofermentans*) colonized the corresponding cheese after 7 to 21 days ripening. Both *Brachybacterium *species also colonized the cheese treated with consortium M, but could only be detected after 81 days, together with the *Brachybacterium *species specific to consortium M (*Br. paraconglomeratum*). Repetition of both treatments revealed the same trends with minor differences including a growth delay (ca. 5 days) for some high-GC species and the additional development of *M. psychrotolerans *at day 20 on the cheese treated with consortium M (data not shown).

**Table 3:** Population dynamics of cheese surface consortia by TTGE^1^

Bacterial species detected with TTGE			Band designation^2^		Consortium F (ripening day)							Consortium M (ripening day)							OMK 704 (ripening day)					
					1	7	14	21	37	81		1	7	14	21	37	81		1	7	14	21	37	81
*Ag. Casei*			x						+	d.						+	d.							
*Al. kapii*			y		d.		+	d.	d.			d.		+	d.	d.							+	
*Br. paraconglomeratum*			m									d.					+							
*Brachybacterium *sp., or *A. arilaitensis*			l		d.	d.	+	d.	d.	d.							+				+	d.	d.	d.
*Br. tyrofermentans*			k		d.			+	d.	d.							+							+
*B. linens*			a, e, g, h, i, n, o		d.	d.	+	d.	d.	d.		d.	d.	+	d.	d.	d.			+	d.	d.	d.	d.
*C. casei*			h, j, v		d.	d.	+	d.	d.	d.		d.	d.	+	d.	d.	d.							
*C. variabile*			b, c		d.	d.		+	d.	d.		d.	d.	+	d.	d.	d.		d.	+	d.	d.	d.	d.
*E. malodoratus*			r				+	d.																
*Lc. lactis*			w (without z')		d.	d.						d.	d.						d.	d.				
*M. psychrotolerans*			w and z'				+	d.															+	
*Mc. gubbeenense*			d		d.			+	d.	d.						+	d.							
*St. equorum, St. epidermidis, or F. tabacinasalis*			q		d.	+	d.	d.	d.	d.		d.	+	d.	d.	d.	d.				+	d.	d.	
*St. equorum*			q and t		d.	+	d.	d.				d.	+	d.	d.						+	d.		
*St. vitulinus*			p		d.	+	d.	d.													+	d.	d.	
uncultured bacterium from marine sediment			f		d.	d.		+	d.			d.	d.			+	d.							+

^1 ^The letter (d.) indicates sampling times where a given species was detected in the TTGE gel. The symbol (+) indicates growth of a species in the smear. Growth was assumed in two cases, i.e. when a band was detected at a sampling time, while absent from the previous sampling time, or when the intensity of the detected band increased, compared to the previous sampling time (valid only at day 7 and 14, i.e. for a 1 log-increase of the total cell count).

^2^The same letter code as for band designation in Figure 3 was used.

### Population dynamics of the defined commercial culture OMK 704 by TTGE fingerprinting

Population dynamics of the defined commercial culture OMK 704 at species level was assessed by TTGE fingerprinting of total DNA extracts (Figure 3, Table 3). All three species of the culture OMK 704 (*C. variabile*, *A. arilaitensis *and *B. linens*) established themselves on cheese surface during the first 14 days. Each of the five *B. linens *strains of the culture OMK 704 exhibited a distinguishable strain-specific TTGE profile (data not shown). The profile of *B. linens *FAM17309 (Bands e, o; Figure 3) was detected in the TTGE fingerprint of day 81 cheese, showing that this strain predominated over other *B. linens *strains at the end of ripening. Additional species not deliberately applied on the cheese colonized the cheese surface along ripening. Two staphylococci species (*St. vitulinus*; *St. equorum*) appeared on day 14 as well as *M. psychrotolerans *and *Al. kapii *on day 37. *Br. tyrofermentans *and an uncultured bacterium from marine sediment completed the high GC community at day 81. Repetition of the treatment revealed the same trends regarding the three defined species. However, the development of non-deliberately applied species was different in the repetition. Three additional species colonized the cheese, i.e. *Enterococcus *sp., *C. casei*, *Ag. casei*, while *Br. tyrofermentans *could not be detected (data not shown).

### *In situ* inhibition of Listeria by complex surface consortia

**Figure 4:**
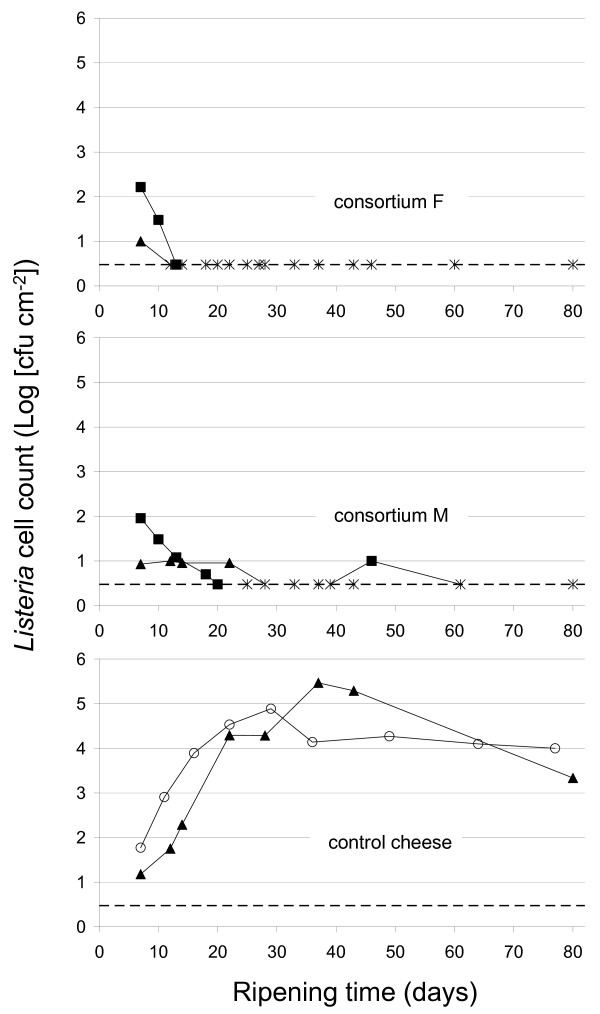
***In situ *inhibition of *Listeria *on cheese surface by complex consortia**. Cheese surfaces were treated with smear brines (3.3% (w/v) NaCl), inoculated with either consortium F, consortium M or the defined commercial culture OMK 704 (control cheese). Two independent experiments were carried out for each treatment. Different symbols indicate different commercial cheese production. Smear brines were inoculated with *Listeria innocua *on day 7 and 8, at 5 × 10^3 ^CFU ml^-1^. Stars indicate times where *Listeria *counts were below the detection limit of the enumeration method (< 3 CFU cm^-2^; dashed line).

Raclette cheeses were artificially contaminated with 4 strains of *Listeria innocua *on day 7 and 8, i.e. when yeasts on cheese surface had reached high counts of 6.5 ± 0.2 × 10^6 ^CFU cm^-2^. From the amount added to the smear brine (5 × 10^3 ^CFU ml^-1^), *Listeria *counts of 1.4 ± 0.9 × 10^1 ^CFU cm^-2 ^(first trial) and of 1.0 ± 0.6 × 10^2 ^CFU cm^-2 ^(repetition) were recovered from the surface immediately after contamination. *Listeria *development was strongly affected by the surface flora applied for ripening. A decrease of *Listeria *counts below the detection limit of the method (< 3 CFU cm^-2^) was observed for cheeses treated with complex consortia F or M supplemented with *Debaryomyces hansenii *FAM14334 (Figure 4). *Listeria *could be recovered from cheese surface (~2000 cm^2^) with an enrichment procedure at the end of ripening (60 to 80 days), for both consortia. In contrast, *Listeria *counts on control cheeses treated with the commercial culture OMK 704 increased to ca. 10^5 ^CFU cm^-2 ^after one month (Figure 4).

## Discussion

To our knowledge, this work describes the first dynamic study of naturally developing anti-listerial cheese surface consortia. The monitoring of two complex consortia obtained from industrial productions was carried out with TTGE, a culture independent fingerprinting technique which enabled species-level detection of high-GC and low-GC bacteria in separate runs.

Previous studies reported a broad range of biodiversity in smear consortia, with 2 to 15 bacterial species detected [2,5,22,23]. High bacterial diversity was observed in consortium F, with 13 species detected at dominant level by culture independent analysis. The cultivation approach detected only 9 of the 13 species present at dominant level in consortium F, but enabled detection of 6 additional species present at subdominant level. TTGE is a semiquantitative approach with limited sensitivity compared to the cultivation approach. However, as fingerprinting technique, TTGE enabled to overcome the arbitrary selection exercised on the flora by the cultivation step, giving a more complete view of biodiversity at dominant level. The combined use of both approaches led to a detailed knowledge of biodiversity in cheese smear flora, as already observed by Feurer *et al*. and Mounier *et al. *[5,24]. The identification strategy used in the present study for the cultivation approach, i.e. all cultivable isolates grouped by TTGE profiles and subsequent sequencing, enabled the detection of intraspecies diversity differentiation in 3 dominant species. This strategy greatly simplified the identification of bands in the TTGE fingerprints of complex consortia corresponding to intraspecies variability. Consortium M displayed slightly less diversity than F with 10 species detected at the dominant level by culture independent analysis.

A total of 20 species were detected in consortia F and M, including eight coryneform bacteria. *C. variabile*, *C. casei*, *B. linens *and *Mc. gubbeenense *are common ripening microorganisms of smear cheeses detected on soft cheeses [5,9] and semi-hard cheeses [2,8,23]. *Br. tyrofermentans *was first isolated from Gruyère cheese [25] and was recently shown to colonize the surface of soft cheeses [5,9]. To our knowledge, this is the first time that *Br. paraconglomeratum *has been detected in cheese although this species has been previously isolated from milk [26]. *Agrococcus casei *was first isolated from Gubbeen, an Irish semi-hard cheese [2]. Three *Staphylococcus *species were isolated in addition to coryneforms. *St. equorum *is common on smear cheeses [6,8,27-29] while *St. vitulinus *was only isolated by Irlinger *et al. *[27] from French cheeses. *St. epidermidis*, a human skin inhabitant, was detected on various Irish semi-hard cheeses [2,8]. Two Gram-positive marine lactic acid bacteria (LAB) and an uncultured bacterium from marine sediment were also part of the dominant flora. *M. psychrotolerans *has been detected in the smear of soft cheeses from Germany and France [5,9]. *Alkalibacterium *sp. was found to be present in many European cheeses including Tilsiter, a semi-hard smear cheese [10]. We also identified potentially undesirable species of enterococci in the subdominant flora of consortium F. Enterococci have a controversial status in the dairy industry. They are considered naturally occurring ripening organisms for artisan Mediterranean cheese [30], but also appear as emerging pathogens due to the virulence factors they tend to harbor [31]. To our knowledge, this study is the first report of the presence of *Facklamia *sp. in cheese. *F. tabacinasalis *was first isolated from powdered tobacco by Collins *et al. *[32] and has recently been detected in raw milk by Delbès *et al. *[33] in a French farm producing Saint-Nectaire cheese and by Hantsis-Zacharov and Halpern [34] in a farm from northern Israel equipped with modern automated milking facilities. The presence of *F. tabacinasalis *on the surface of smear cheese may constitute a health hazard, as this species was shown to be α-haemolytic on horse blood [32]. Moreover, from six *Facklamia *species described to date, four were isolated from human clinical specimen [35].

We observed highly similar microbial community structures of consortia F and M, with 9 species being common to both consortia at dominant level, despite different ripening procedures. High interbatch diversity was described by Rea *et al. *[8] in a single cheese ripening facility of Gubbeen, an Irish semi-hard smear cheese, over 8 years production, which may be related to a lack of humidity and temperature control during ripening of Gubbeen cheese. In the present study, the production of Swiss Raclette type cheese with defined production and ripening parameters led to the development of a similar flora in two distinct dairies. The source of this highly diverse flora remains unidentified but possible sources could be the brine bath, skin of the workers or wooden shelves, as shown by Mounier *et al. *[36] for Gubbeen cheese.

The high biodiversity is particularly surprising in the case of dairy F, where the smear brine is freshly prepared prior to each smearing and inoculated with a defined ripening culture of only 3 bacterial species. Moreover, the smear brine is applied by a cheese ripening robot that smears the young cheeses first. However, the microflora of the brine bath is not controlled and might be one of the major sources. In particular, the brine bath (18-22% (w/v) NaCl) could be suitable to maintain the two halophilic and alkaliphilic marine LAB detected in consortium F, as some strains of *M. psychrotolerans *and *Al. kapii *were shown to grow at salt concentration as high as 21% (w/v) by Ishikawa *et al. *[37,38].

Dynamic studies of consortia F and M inoculated at same cell counts on cheese surface revealed a similar sequential development of nine bacterial species, i.e. *Lc. lactis, St. equorum, Al. kapii, C. casei*, *B. linens, C. variabile*, an uncultured bacterium from marine sediment, *Mc. gubbeenense *and *Ag. casei*. The development of this microbial community prevented growth of *Listeria innocua*, inoculated at 5 × 10^3 ^CFU ml^-1 ^smear brine on cheeses at day 7 and 8, over 60 to 80 days ripening. Contamination at day 7 and 8, i.e. when yeasts reached their highest density, provided optimal growth conditions for *Listeria*, as shown by the rapid *Listeria *growth on control cheese. Strong antilisterial activities were shown in this unfavorable condition for consortia F and M. Antilisterial activities of complex undefined cheese surface consortia were already observed in previous studies [9,15]. Maoz *et al. *[9] reported a total inhibition of *L. monocytogenens *during 40 days ripening of a soft smear cheese with an initial contamination level of 1.6 × 10^3 ^CFU ml^-1 ^smear brine.

The surface of smear cheese contains a limited range of substrates supporting growth of microorganisms, mainly lactose and lactate. Lactose is mostly metabolized by LAB during curd acidification and initial ripening. The residual lactose can be metabolized on the cheese surface by yeasts during the first days of ripening, as shown for soft cheeses by Leclercq-Perlat *et al. *[39]. Lactate metabolized by yeasts into CO_2 _and H_2_O leads to deacidification of the cheese surface [40]. As a result, lactate continuously diffuses from the core to the surface of the cheese. Lactate can be totally consumed by surface microorganisms in soft cheeses [41]. Several smear bacterial species, i.e. *Brevibacterium aurantiacum*, *C. casei*, *C. variabile*, *Mc. gubbeenense *and *St. saprophyticus*, were shown to use lactate and casaminoacids for growth [42]. In contrast, *Listeria *sp. can only use a limited range of carbon sources for growth, including glucose, glycerol, fructose and mannose, while no growth occurs on lactate or casaminoacids [43-46]. Premaratne *et al. *[44] showed that *Listeria monocytogenes *may utilize alternative carbon sources, such as N-acetylglucosamine and N-acetylmuramic acid, which are major components of bacterial and fungal cell walls [44,47]. In addition, the yeast cell wall contains a mannan glycopeptide with mannose [48], a sugar metabolized by *Listeria *sp. *Listeria *growth on smear cheese can therefore be limited by a low availability of carbon source and stimulated by components of smear microorganisms.

Marine LAB ferment glucose into lactate and assimilate mannose [37,38]. Ishikawa *et al. *[38] reported that *Al. kapii *can utilize a fairly limited range of carbon sources. In the present study, *M. psychrotolerans *and/or *Al. kapii *established early on cheeses treated by complex consortia, i.e. between day 14 and day 20. We believe competition for nutrients between marine LAB and *Listeria *sp. may be involved in *Listeria *inhibition in the smear since the development of *M. psychrotolerans *and *Al. kapii *occurred simultaneously with the decrease of *Listeria *counts for both cheeses treated with consortium F (first trial and repetition) and for one cheese treated with consortium M (repetition). In addition, *Listeria *growth on control cheese stopped when *M. psychrotolerans *and *Al. kapii *were first detected in the smear, i.e. on day 37. Hain *et al. *[49] reported a microarray experiment conducted with the antilisterial complex smear consortium described by Maoz *et al. *[9]. Genes involved in energy supply were mostly up-regulated after 4 hours of contact between *Listeria monocytogenes *and the consortium, suggesting that *Listeria *had entered a state of starvation. While Maoz *et al. *[9] detected *M. psychrotolerans *in the aforesaid smear consortium by cultivation methods, they may have overlooked the presence of *Al. kapii *or related species.

## Conclusions

This work reports the first study of population dynamics of antilisterial cheese surface consortia. Dynamics of two consortia obtained from industrial productions revealed highly similar, with the sequential development of 9 common species, whereas development of both consortia inhibited *Listeria *growth over the whole ripening period. Next to common cheese surface bacteria, the two consortia contained marine lactic acid bacteria (LAB) that developed early in ripening, shortly after the growth of staphylococci and concomitant with a decrease in *Listeria *cell counts. Competition for nutrients between marine LAB and *Listeria *sp. could be involved in the observed inhibition. Temporal temperature gradient gel electrophoresis revealed decisive to detect all marine bacteria present at dominant level in the smear, as only one of three species was detected by the culture dependent approach. Further cheese ripening experiments are needed to investigate the potential contribution of marine LAB to antilisterial activity.

## Methods

### Collection of cheese surface consortia and microbial cultures

Cheese surface consortia were collected from two Swiss cheese manufacturers of Raclette type cheese made of pasteurized milk. Consortium F was collected from a 4-weeks old cheese produced with a defined surface ripening culture in industrial-scale dairy F. The surface ripening culture was composed of OFR9 (Danisco A/S, Copenhagen, Denmark), containing *Brevibacterium linens*, *Brevibacterium casei *as well as three yeasts, and OMK 703 (Research Station Agroscope Liebefeld-Posieux ALP, Bern, Switzerland), containing *Brevibacterium linens*, *Arthrobacter arilaitensis *as well as two yeasts. Consortium M was collected from a 6-weeks old cheese in small-scale dairy M, where the cheeses were treated with old-young smearing, with a smear brine derived from Gruyère type cheese. Surface consortia were scraped off the cheese (~2000 cm^2^; ~10 g), homogenized in a stomacher in 100 ml 3.3% (w/v) NaCl for 4 min and stored at 4°C until further use but not longer than 30 days. Long-term storage (up to 7 months) was carried out by addition of 20% glycerol and freezing at -30°C. The commercial surface culture OMK 704 (ALP, Bern, Switzerland), used as control in cheese ripening experiments, contained one yeast (*Debaryomyces hansenii *FAM14334, ALP culture collection), five *Brevibacterium linens*, five *Corynebacterium variabile*, and one *Arthrobacter arilaitensis *strains. Each strain of the commercial culture was provided in a liquid form and stored at 4°C (short term) or at -30°C with addition of 20% glycerol (long term). For safety reasons, non pathogenic *Listeria *strains were used as a model for *L. monocytogenes *in cheese ripening experiments. *Listeria innocua *80945-8, 81000-1, 81003-3, and 81587-4 (Laboratory of Food Biotechnology, ETH Zurich, Zurich, Switzerland) had previously been isolated from smears by ALP (Bern, Switzerland). *Listeria *strains were grown in tryptic soy broth (Oxoid, Pratteln, Switzerland) supplemented with 0.6% (w/v) yeast extract (Merck, Dietikon, Switzerland) at 30°C for 14 h.

### Cell enumeration of cheese surface consortia

Total cell counts were determined on TGYA (Tryptic Glucose Yeast Agar, Biolife, Milano, Italy) supplemented with 1% (w/v) casein peptone (BBL, Heidelberg, Germany) after incubation at 30°C for 3 days, followed by incubation at room temperature with daylight exposure for another 7 days. Staphylococci were counted on BP agar (Baird Parker RPF agar; BioMérieux, Geneva, Switzerland) and MSA (Mannitol Salt Agar, Oxoid, Pratteln, Switzerland) after incubation at 37°C for 6 days. Yeast counts and mould counts were both determined on PY agar (Phytone Yeast extract agar, BBL, Heidelberg, Germany) incubated at 30°C. The plates were examined after 3 days for yeasts and after 6 days for moulds. Enterococci were determined on KFS agar (KF *Streptococcus *agar, Becton Dickinson AG, Allschwil, Switzerland) incubated at 42°C for 3 days, and *Listeria *on Palcam agar (Oxoid, Pratteln, Switzerland) incubated at 37°C for 2 days, all under aerobic conditions. Lactic acid bacteria were counted on MRS agar with Tween 80 (De Man *et al.*, 1960, Biolife, Milano, Italy) incubated at 37°C for 6 days, under anaerobic conditions which were generated using GENbox anaerobic systems (Biomérieux, Geneva, Switzerland). At the end of ripening, the presence or absence of *Listeria *was assessed using a three-step enrichment procedure that was previously validated against the reference method ISO 11290-1 for use on smear samples by ALP (Bern, Switzerland). 10 g (~2000 cm^2^) of smear were homogenized in 90 g tryptic soy broth supplemented with 0.6% (w/v) yeast extract, 0.02% (w/v) Delvocid^® ^(DSM, Heerlen, Netherlands), 0.001% (w/v) acriflavin (Fluka, Buchs, Switzerland), and 0.004% (w/v) nalidixic acid (Fluka, Buchs, Switzerland) for 4 min using a Stomacher and incubated at 30°C for 24 h. After this step, 1% (v/v) of enriched sample was inoculated to supplemented tryptic soy broth and incubated again at 30°C for 24 h. Presence or absence of *Listeria *was then checked by streaking a loopful of the second enrichment media on ALOA agar (Biolife, Pero, Italy) that was incubated at 37°C for 24 h.

### DNA extraction of complex consortia and single isolates

Total DNA extraction of cheese surface consortia was carried out with 1 ml homogenate containing 10^7 ^to 10^9 ^CFU ml^-1 ^that was centrifuged at 18'000 × g for 5 min. The resulting pellet was stored at -20°C until further use. The DNA extraction protocol was modified from Chavagnat *et al. *[50]. The frozen pellet was resuspended in 1 ml 0.1 M NaOH, incubated at room temperature for 15 min and centrifuged at 18'000 × g for 5 min. The pellet was resuspended in 1 ml TES buffer (10 mM EDTA, 0.1. M tris(hydroxymethyl)-aminomethane, 25% (w/v) saccharose) containing 0.25% (w/v) lysozyme (50000 U mg^-1^, Merck, Dietikon, Switzerland), incubated at 37°C for 1 h, and centrifuged at 18'000 × g for 5 min. The pellet was resuspended in 190 μl G2 Buffer (EZ1 DNA Tissue Kit, Qiagen, Basel, Switzerland) and 10 μl proteinase K (EZ1 DNA Tissue Kit; Qiagen, Basel, Switzerland) were added. This suspension was incubated at 56°C for 1 h after which DNA was further purified by BioRobot^® ^EZ1 (Qiagen, Basel, Switzerland) and analyzed by TTGE, as described below.

DNA extraction of single isolates was carried out by dissolving one colony of a pure culture in 0.2 ml tris-K buffer (0.01 M tris(hydroxymethyl)-aminomethane (Merck, Dietikon, Switzerland)) containing 0.5 μl ml^-1 ^Tween 20 (Fluka, Buchs, Switzerland) and 0.24 mg ml^-1 ^proteinase K (Sigma-Aldrich, St. Louis, USA). This suspension was first heated at 60°C for 1 h followed by 95°C for 15 min, and centrifuged at 10'000 × g for 5 min resulting in a DNA containing supernatant that was further analyzed by TTGE.

### Temporal temperature gradient gel electrophoresis (TTGE)

PCR amplification of the V3 region of the 16S rDNA (~200 bp) was performed according to Ogier *et al. *[12] using a Biometra T-Personal thermocycler (Biometra, Göttingen, Germany) with direct amplification using primers HDA1-GC and HDA2 (Microsynth, Balgach, Switzerland) and ~100 ng of bacterial DNA. Ten μl of PCR products were separated on a 2% (w/v) agarose gel to check successful amplification with a molecular weight standard of TriDye 100 bp DNA Ladder (BioConcept, Allschwil, Switzerland). TTGE analysis was carried out as described by Ogier *et al. *[12] with the following modifications. The electrophoresis was run in 1.5 × TAE buffer (1.5 mM EDTA, 60 mM tris(hydroxymethyl)-aminomethane, 60 mM acetic acid) at 65 V for 16 h, with a temperature ramp of 0.3°C h^-1 ^from 66 to 70°C. The gel concentrations were optimized to enable visualization in separate runs of high-GC bacteria (8 M urea; 8.5% (w/v) acrylamide (37.5:1)) and low-GC bacteria (7 M urea; 8% (w/v) acrylamide (37.5:1)) by empirical approach using a ladder of dairy bacteria harboring a wide range of GC-contents (from 49% for *Lactobacillus plantarum *to 60% for *Propionibacterium *sp.). Volumes of 20 μl (isolates) or 30 μl (complex consortia) of PCR products were mixed with 20 μl loading dye (0.25% (w/v) Orange G, 50% (w/v) sucrose; Fluka, Buchs, Switzerland) and loaded in each well. The detection limit of the method proved similar to Ogier *et al. *[12], with detection of bacterial species accounting for at least 1% of the total DNA amount.

### Identification of single isolates by partial sequencing of 16S rDNA

Groups of isolates with identical TTGE profiles were formed and a representative isolate of each group was selected for further 16S rDNA sequencing analyses. A 1400-bp fragment of the 16S rDNA was amplified with universal primers 16SUNI-L and 16SUNI-R [51]. The 50-μl reaction mixture contained ~20 ng DNA (NanoDrop^® ^ND-100, Witec AG, Littau, Switzerland), 2.5 U of Taq DNA polymerase (Euroclone, Pero, Italy), 0.4 μM of each primer (Microsynth, Balgach, Switzerland), 200 μM of each deoxynucleoside triphosphate (Amersham Biosciences, Otelfingen, Switzerland), and the reaction buffer (Euroclone, Pero, Italy) consisting of 10 mM Tris-HCl, 50 mM KCl, and 1.5 mM MgCl_2_. The amplification was performed in a Biometra T-Personal thermocycler (Biometra, Göttingen, Germany) with the following temperature profile: 94°C for 3 min, 35 cycles of 94°C for 30 s, 54°C for 30 s, 72°C for 60 s, and a final annealing at 72°C for 7 min. Amplified DNA was purified using the GFX-PCR DNA Purification Kit (GE Healthcare Biosciences, Otelfingen, Switzerland). Partial sequencing was carried out with primer 16SUNI-L and the BigDye^® ^Terminator v1.1 cycle sequencing kit (Applied Biosystems, Rotkreuz, Switzerland) and analyzed in an ABI Prism 310 genetic analyzer (Applied Biosystems, Rotkreuz, Switzerland). Species identification was obtained by matching the obtained partial sequence (500 to 900 bp) to deposited sequences in the GenBank public database using the BLAST program.

### Identification of TTGE bands by partial sequencing of the 16S rDNA

Bands of the complex TTGE fingerprints that could not be identified by comparison with the database were excised, cloned and sequenced as described by Ogier *et al. *[12]. The eluted DNA was amplified by PCR using primers HDA1 and HDA2 (Microsynth, Balgach, Switzerland). PCR products were purified using the GFX-PCR DNA Purification Kit (GE Healthcare Biosciences, Otelfingen, Switzerland), ligated into pGEM^®^-T Easy vector (Promega, Dübendorf, Switzerland) and transformed into *Escherichia coli *(Subcloning Efficiency™ DH5™ Competent Cells, Invitrogen, Basel, Switzerland). After plasmid purification, the insert was amplified by PCR with primers HDA1-GC and HDA2. The PCR product was analyzed by TTGE to confirm its position in the gel and sequenced from both sides with primers HDA1 and HDA2. The sequence obtained (~200 bp) was matched to deposited sequences in the GenBank public database.

### Cheese ripening experiments

Raclette type cheeses (~6 kg; 2000 cm^2^) produced from pasteurized milk in dairy F were taken immediately after brining. A water content of 44.9% (w/w) and salt content of 1.8% (w/w) were measured in a 24 h-old cheese from the production batch, by gravimetric analysis (ISO 5534/IDF 4:2004) and by potentiometric titration (IDF Standard 88A:1988), respectively. Cheeses were ripened in a pilot plant cheese cellar with controlled temperature at 11°C and relative humidity at 95% for 2 to 3 months. Cheeses were smeared daily until day 15 and twice a week thereafter, using 20 ml smear brine (3.3% (w/v) NaCl) per cheese side. Three different treatments were applied on cheeses and two independent experiments were carried out for each treatment. Cheeses were treated with 20 ml of smear brines inoculated with 5 × 10^8 ^CFU ml^-1 ^of either: consortium F, consortium M or the commercial culture OMK 704. In addition, 1 × 10^7 ^CFU ml^-1 ^of the yeast strain Debaryomyces hansenii FAM14334 were inoculated in all smear brines. Smear brines were prepared fresh before each smearing with the following protocol. The appropriate amounts of consortium or defined culture and yeast were added in a 50 ml centrifugation tube and the volume was adjusted to 20 ml by addition of 3.3% (w/v) NaCl. Tubes were then centrifuged at 5'000 × g for 15 min, and the pellet was resuspended in 20 ml of fresh 3.3% (w/v) NaCl.

Cheeses were artificially contaminated twice with *Listeria *after 7 and 8 days ripening. *Listeria *inoculum was prepared as follows. Overnight cultures of 4 *Listeria innocua *strains were mixed in a 1:1:1:1 ratio, diluted 10'000 times in 0.9% (w/v) NaCl, and 0.3 ml of the dilution were added to each smear brine after the centrifugation step, to reach a concentration of ca. 5 × 10^3^ CFU ml^-1^. The brushes and wooden shelves were washed and autoclaved prior to each use. Wooden shelves were first changed after one week and every three weeks thereafter. The pH of the cheese surface was periodically measured *in situ *using a flat membrane electrode (InLab^® ^Surface, Mettler-Toledo, Greifensee, Switzerland).

### Microbial analyses of cheese surface during ripening experiments

Approximately 25 cm^2 ^of cheese surface were scraped off using sterile cotton rolls (IVF Hartmann, Neuhausen, Switzerland) and aseptically transferred into a stomacher bag. Each sample was suspended in 25 ml pre-heated (45°C) peptone water, composed of 1% (w/v) casein peptone, 0.5% (w/v) NaCl and 2% (w/v) tri-sodium citrate dehydrate, all from Merck (Dietikon, Switzerland), and homogenized for 4 min using a Stomacher (Silver Masticator; IUL Instruments GmbH, Königswinter, Germany). 1 ml of this solution was submitted to total DNA extraction for TTGE as described above. Serial dilutions in 0.9% (w/v) NaCl were prepared and plated on TGYA, PY agar and Palcam agar. At the end of ripening, 10 g of smear were harvested and tested for the presence of *Listeria *using an enrichment procedure as described above.

## Authors' contributions

ER carried out the experiments, evaluated the results and drafted the manuscript. MH participated in the creation of the TTGE database and in the repetition of the cheese experiment. SMS and EEM participated in the conception and coordination of the study and revision of the manuscript. CL provided guidance during the whole study and revised the manuscript. All authors read and approved the final manuscript.
